# Towards smart sterile supply cycles

**DOI:** 10.1007/s11548-026-03700-5

**Published:** 2026-05-20

**Authors:** Ha Uyen Nguyen, Lea Kristin Stoll, Klaus Radermacher, Jan Heibeyn

**Affiliations:** Chair of Medical Engineering (mediTEC), RWTH Aachen University, Aachen, Germany

**Keywords:** Sterile goods, CSSD, Smart hospital, SDC

## Abstract

****Purpose**:**

The sterile supply cycle (SSC) is fundamental to perioperative care but remains fragmented and largely operated as an open-loop process in which deviations are often detected only in the operating room (OR). Current smart hospital concepts mainly address patient-centred systems and insufficiently integrate perioperative material logistics and sterile supply processes. This work proposes a conceptual framework for integrating ORs, OR management, and the SSC into a unified smart hospital network in order to enable end-to-end visibility and data-driven coordination.

****Methods**:**

A staged, model-driven approach was applied, combining preliminary empirical studies with principles from industrial smart manufacturing. An online survey and benchmarking data identified recurring systemic weaknesses, including missing or defective instruments and weak OR–CSSD information coupling. These findings were translated into functional, technical, and architectural requirements, which were iteratively refined through expert discussions.

****Results**:**

The resulting framework adopts a service-oriented, layered architecture comprising perception, network, gateway, knowledge, and application layers. It represents ORs, OR management, and the CSSD as interoperable but autonomous subsystems and outlines an event-driven integration logic for cross-domain data exchange, traceability, and future decision support.

****Conclusions**:**

This work presents a conceptual integration framework for embedding the SSC within broader smart hospital architectures. By making sterile supply processes visible as an interoperable subsystem and by structuring cross-domain information flows, it provides a foundation for future prototyping and evaluation of more coordinated and resilient sterile supply processes.

## Introduction

The trend towards smart hospitals includes increasingly connected, data-driven, and automated clinical processes, infrastructure, and information systems [1, 2]. These concepts aim to improve patient safety and quality of care while using scarce resources more efficiently [3]. In perioperative care, however, such goals cannot be achieved by focusing on the operating room (OR) alone. Surgical readiness also depends on the sterile supply cycle (SSC), that is, the coordinated progression of instruments from intraoperative use through reprocessing and storage to timely re-provisioning. The performance of this cycle directly affects patient safety as well as the effectiveness and efficiency of the operating theatre [4–6]. Current smart hospital concepts and interoperability approaches, including IEEE 11073 Service-oriented Device Connectivity (SDC) [7] and clinical IoT platforms, predominantly target devices, information systems, and assistance functions within the OR. By contrast, the associated material logistics and sterile supply processes, typically coordinated through the Central Sterile Supply Department (CSSD), remain insufficiently represented as an integrated part of smart hospital architectures. This is notable because the OR is one of the most resource-intensive hospital areas, and its schedules depend on the timely availability of staff, rooms, anaesthesia, and sterile devices [8]. Delays in material provision can trigger rescheduling, intraoperative disruptions, additional transports, or cancellations, with significant costs and risks to patient safety [9, 10]. Against this background, this paper proposes a conceptual framework for integrating ORs, OR management, and the CSSD into a unified smart hospital network. Rather than presenting a validated production architecture, the paper aims to structure the requirements, information flows, and architectural principles needed to represent the SSC as an interoperable and data-enabled subsystem within the broader smart hospital.

## Preliminary studies

To ground the proposed concept in observed practice, the following preliminary studies are used to identify recurring operational deficits and derive requirement patterns relevant to SSC integration.

Two preliminary studies in Germany highlight systemic weaknesses in the SSC. A survey among OR and CSSD staff [11] showed that OR personnel (6 OR managers, 2 nurses) perceived late or missing instrument sets, missing or defective instruments, and the need to search for replacements as the most influential problems from a suggested list of challenges, while CSSD managers (n=5) emphasise staff shortages, high workloads, and insufficient support as primary challenges. These perceptions are consistent with benchmarking data from 48 CSSDs collected between 2023 and 2024 [12], where 46 % of departments reported frequent OR complaints about defective instruments and 42 % about missing or incorrect items in instrument sets. Together, the results suggest an operationally critical yet structurally fragile SSC in which process deviations often become visible only at the point of use in the OR, reflecting a largely open-loop process where quality and planning errors are detected late and corrected under the most disruptive and costly conditions.

Further analysis of the benchmarking and workflow data indicates that these issues are rooted in structural deficits in process design and information flow [11, 12]. CSSD managers primarily attributed complaints to personnel shortages, high workloads, device errors, and communication problems with the OR. In many departments, work organisation still follows a first-in–first-out (FIFO) logic with only weak coupling to the dynamic priorities of the upcoming surgical schedule. Workflow analyses conducted in seven German hospitals (2020–2025) show a wide range of maturity levels—from purely FIFO-based processing without visibility into upcoming OR demand to digitally triggered set production based on OR planning data. Even in the latter, frequent urgent requests and short-notice changes remain common. At the same time, documentation and planning in many hospitals continue to rely on paper lists, stand-alone software, or vendor-specific tracking solutions. Where digital tools exist, they are often proprietary, use inconsistent data models, and connect to hospital IT via custom interfaces. Consequently, SSC processes are frequently not represented in a consistent, machine-interpretable way, and there is often no unified mechanism to communicate SSC status or events to clinical applications or OR management systems. The result is a structurally reactive mode of operation in which the SSC is expected to absorb growing volatility in surgical schedules, while OR–CSSD information loops remain largely manual, asynchronous, and fragmented.

From a smart hospital perspective, these findings indicate that timely, semantically consistent, and cross-domain available data are central prerequisites for more resilient sterile supply processes. In particular, the persistent gap between internal CSSD data, such as staffing, machine states, queues, and quality checks, and OR-related information, such as case schedules, case priorities, and expected instrument demand, limits the ability of current digital systems to support clinical needs in a coordinated manner. Concepts for process and production control in the CSSD, partly inspired by industrial shopfloor management, suggest several functional directions for improvement, including demand-oriented packing based on real-time OR information, adaptive digital worklists, machine and set status dashboards, and standardised escalation mechanisms. In this paper, such examples are not treated as validated solution modules, but as requirement-oriented illustrations of the type of coordination capabilities that an integrated SSC architecture should support.

## Related works

Smart hospitals aim to support safer and more efficient care through connected, data-driven, and increasingly automated clinical processes, infrastructure, and information systems [1, 3]. Relevant work for the present paper spans three adjacent domains: smart hospital interoperability architectures, CSSD-specific digitalisation approaches, and smart manufacturing concepts as a source of transferable design principles.

Several architectural frameworks have been proposed to support data integration and interoperability in smart hospital environments. For example, Yadegari and Asosheh [13] describe a layered IoT architecture comprising data acquisition, networking, gateway, knowledge, and application functions, while Martens et al. [14] report large-scale integration of medical devices and hospital information systems using standards such as HL7, DICOM, and FHIR. These works demonstrate the potential of structured, interoperable data architectures in clinical environments. However, they mainly focus on patient-facing systems, medical devices, and clinical information flows. Perioperative material logistics and the CSSD are not represented as an explicit, interoperable subsystem within the broader smart hospital architecture.

To address cost pressures, logistical complexity, and patient safety risks in instrument reprocessing, CSSDs increasingly employ technical and operational solutions, most notably instrument tracking and identification systems based on RFID, optical codes, or physical markings [15]. Commercial planning and management solutions are also used to support inventory control and local process coordination. However, such systems often remain stand-alone applications with proprietary data models, limited interoperability, and only weak coupling to OR workflows and hospital-wide coordination mechanisms [16]. Existing CSSD digitalisation therefore improves traceability and local management, but rarely establishes end-to-end, machine-interpretable integration between CSSD processes, OR management, and clinical applications.

In the industrial domain, smart manufacturing refers to the integration of data networking and information technology into physical production processes in order to enable more efficient, flexible, and responsive operations [17]. Architectures in this domain typically combine planning, execution, event logging, and interoperable machine connectivity, for example through Advanced Planning and Scheduling (APS), Manufacturing Execution System (MES), process mining, and open communication standards such as Open Platform Communications Unified Architecture (OPC UA) [18–21]. These concepts are relevant because the SSC likewise operates under volatile demand, constrained resources, and strict quality and traceability requirements. However, smart manufacturing does not directly address the clinical, organisational, and safety-critical conditions of perioperative sterile supply. In this paper, it is therefore used as a source of design principles rather than as direct prior art.

In summary, current smart hospital architectures show how interoperable clinical data infrastructures can be designed, but they largely neglect the CSSD as an integrated subsystem. Existing CSSD technologies address local tracking and planning needs, yet typically remain proprietary and weakly connected to OR coordination. Smart manufacturing offers useful architectural principles for closed-loop, event-driven coordination, but not a clinically grounded framework for sterile supply integration. This leaves a gap at the intersection of these three domains, which the present paper addresses by proposing a conceptual framework for integrating ORs, OR management, and the CSSD into a unified smart hospital network.

## Conceptual framework

This section presents a conceptual framework for integrating the CSSD into a broader smart hospital architecture. The framework was developed using a staged, model-driven process intended to derive and structure architectural principles. First, we generated an overview of current SSC processes based on our preliminary studies (survey, benchmarking, workflow analyses) and a targeted review of scientific and technical literature on CSSD workflows, traceability, and international SSC approaches. In parallel, we examined interconnectivity and technical boundary conditions in the operating room (OR) as the primary downstream user of sterile goods. The resulting requirements were clustered into functional, technical, architectural, and risk-related categories. Alternative architectural patterns were then discussed with domain and technical experts, including five software developers, one CSSD manager, one OR manager, and two managing directors of SSC software providers, in order to assess their interoperability potential, conceptual feasibility in typical hospital environments, alignment with observed user needs, and anticipated practical benefits.

The proposed framework follows a service-oriented architecture approach in which devices and software modules expose their capabilities through well-defined service and event interfaces. Comparable principles have been applied in industrial automation through technologies such as Open Platform Communications Unified Architecture (OPC UA), where they have shown advantages in modularity and system extensibility. In the hospital context, ISO/IEEE 11073 Service-oriented Device Connectivity (SDC) appears particularly relevant for interoperable device communication in the perioperative environment. Within this framework, it is therefore considered a promising mechanism not only for device integration within the OR, but also as a potential basis for extending interoperability to adjacent perioperative domains such as the CSSD, where compatible devices and interfaces are available. At a conceptual level, the framework adopts the five architecture layers introduced in smart hospital research [13] in order to structure functions and information flows:

- The **Perception Layer** comprises the acquisition of data from heterogeneous sources, including CSSD devices such as washer–disinfectors as well as user-generated inputs, for example the manual reporting of defective or missing instruments during assembly.
- The **Network Layer** covers the communication protocols and transmission technologies used for data exchange across the system. In the present framework, this layer is deliberately kept technology open in order to accommodate different technical conditions and future developments.
- The **Gateway Layer** is responsible for harmonising incoming data and establishing system-wide consistency, including preprocessing steps to standardise heterogeneous input formats. It also includes conceptual provisions for persistent storage, for example through databases or data warehouse structures, in order to support historical analysis and traceability.
- The **Knowledge Layer** contains functions for planning, optimisation, forecasting, and decision support. It is intended to use integrated process data to support more informed operational decisions, for example through predictive models or process-monitoring mechanisms.
- The **Application Layer** comprises the user-facing systems that support operational and planning activities, such as OR scheduling tools or CSSD management applications.

**Fig. 1:**
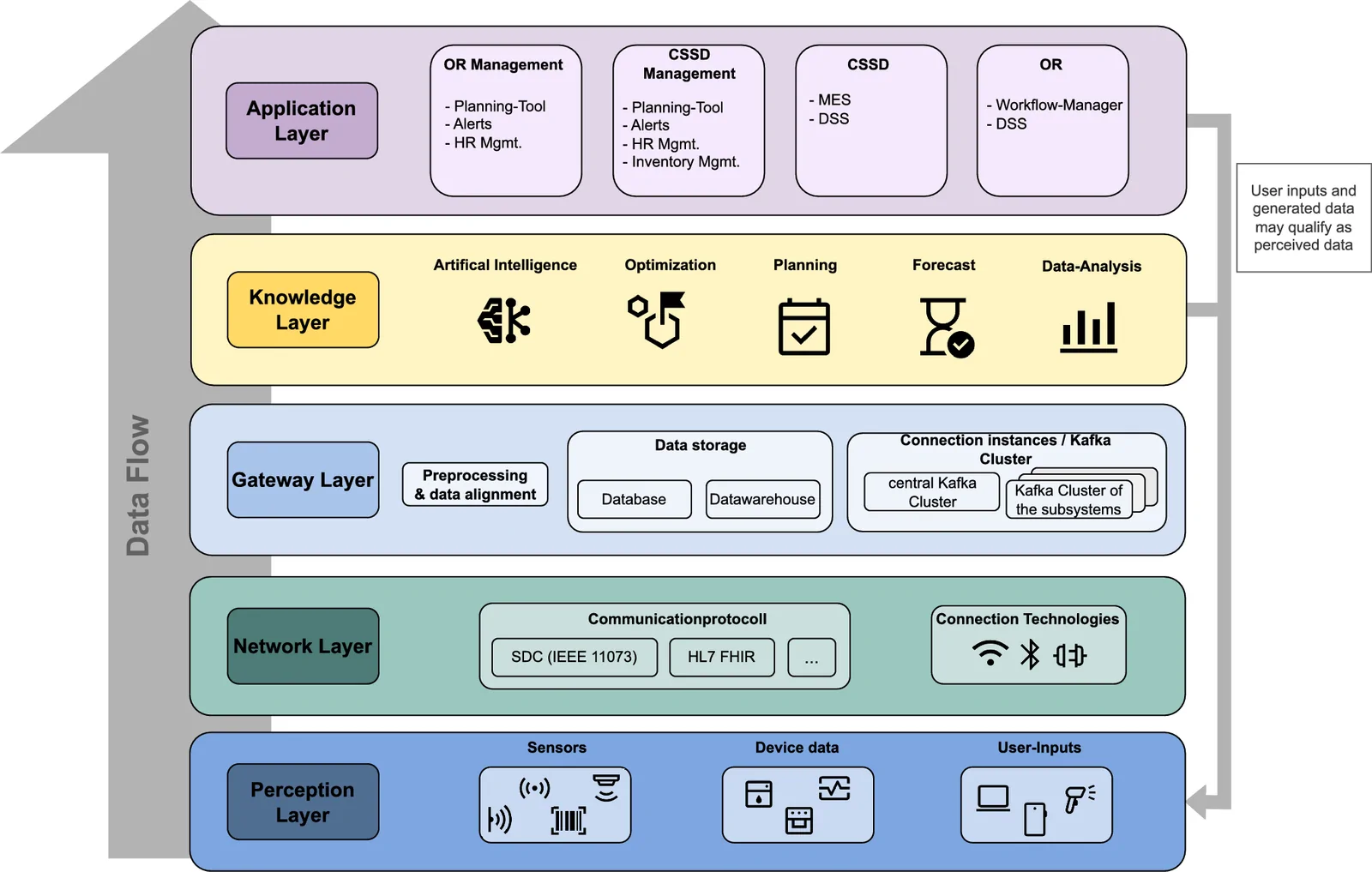
Illustration of the architectural layers and their associated functionalities in the system

An overview of the architecture and the main functions assigned to each layer is provided in Fig. 1.

The conceptual framework primarily assumes a bottom-up information flow, in which data are generated at lower layers and made available to higher layers for aggregation, interpretation, and use. This supports a structured progression from data acquisition to operational and analytical use. An important exception concerns the Application and Knowledge Layers, in which new information may also be generated. Users may, for example, enter or modify planning data in the Application Layer, such as the OR schedule, which in turn affects optimisation and forecasting functions. Likewise, outputs generated in the Knowledge Layer may influence planning decisions and trigger responses in connected systems. For instance, the prediction that a required sterilised instrument set will not be available in time for a scheduled surgery may trigger proactive escalation or replanning mechanisms.

To operationalise the framework, the relevant actors and their roles are represented as follows:

- The **OR** uses instrument sets and records their use. Information on instrument set utilisation and estimated procedure duration, derived from OR systems and, where available, SDC-based device and workflow data, is required to determine when instrument sets can be released for return, reprocessing, and transport.
- **OR Management** is responsible for surgical planning and maintains the OR schedule, which also defines expected sterile goods demand. Within the framework, this subsystem may receive alerts when factors such as instrument availability or device defects threaten schedule feasibility. Personnel planning is also relevant at this level, as staffing constraints in both the OR and CSSD influence whether the surgical schedule can be executed as planned.
- **CSSD Management** oversees reprocessing operations and includes planning and personnel management functions intended to align instrument set processing with OR demand. It may also receive alerts when sterilisation requirements are not expected to be met. In addition, inventory-related functions are relevant in order to support timely repair or replacement of defective instruments.
- The **CSSD** is represented as a subsystem that should support manufacturer-independent communication. SDC is considered the preferred standard where applicable, while non-SDC-compatible devices and stations must also be integrated. Processing stations such as washer–disinfectors, sterilisers, and assembly stations can therefore be represented through interoperable interfaces and connected via an integration component. Forecasting functions may additionally be incorporated in order to estimate instrument reprocessing times.

These actors are represented as independent but connected subsystems. Although the framework allows for further extensions within a broader smart hospital environment, the present work focuses on the overarching integration concept rather than on the detailed implementation of individual functions.

Direct communication between individual subsystems is not sufficient for support functions that depend on information from multiple domains, such as OR scheduling support or CSSD plan optimisation. For this reason, the framework introduces a central integration instance that mediates inter-system communication. Conceptually, this integration instance is intended to decouple message producers and consumers, support translation between protocols and data formats, and enable persistent event logging for traceability and later analysis. It may also provide a shared, temporally aligned data basis for planning, monitoring, and decision support functions.

As a reference technology for this integration logic, Apache Kafka (hereafter Kafka) is considered a plausible option because it supports event-driven communication, decoupling of data producers and consumers, and persistent message storage [22]. In the present paper, however, Kafka is introduced as an illustrative implementation option within the conceptual framework rather than as a validated final design choice. Its potential suitability for specific clinical requirements, including latency, fault tolerance, cybersecurity, and operational cost, remains to be evaluated in future implementation work. However, Kafka has already been used and studied in high-demand financial settings such as fraud detection and anti-money-laundering pipelines, where recent work highlights its role in large-scale, real-time transaction processing [23]. A connected data warehouse may further provide a consolidated and temporally aligned data basis for optimisation and decision support modules. Kafka is also compatible with data-streaming pipelines for machine-learning-based analytics and inference, which may become relevant in future extensions of the framework [22].

To further support modularity and subsystem autonomy, one possible realisation of the proposed integration logic is a hierarchical structure consisting of a central Kafka cluster and local clusters within individual subsystems. In such a setup, local clusters would allow internal processes to remain decoupled and operationally independent, while the central cluster would aggregate selected cross-subsystem information for planning, monitoring, and analytical applications. Synchronisation between clusters could be achieved through controlled cross-cluster replication of selected topics or data streams. Conceptually, this would allow subsystem autonomy to be preserved while enabling a shared cross-domain data foundation. At the present stage, however, this hierarchical structure should be understood as a proposed design option that requires future technical evaluation.

**Fig. 2:**
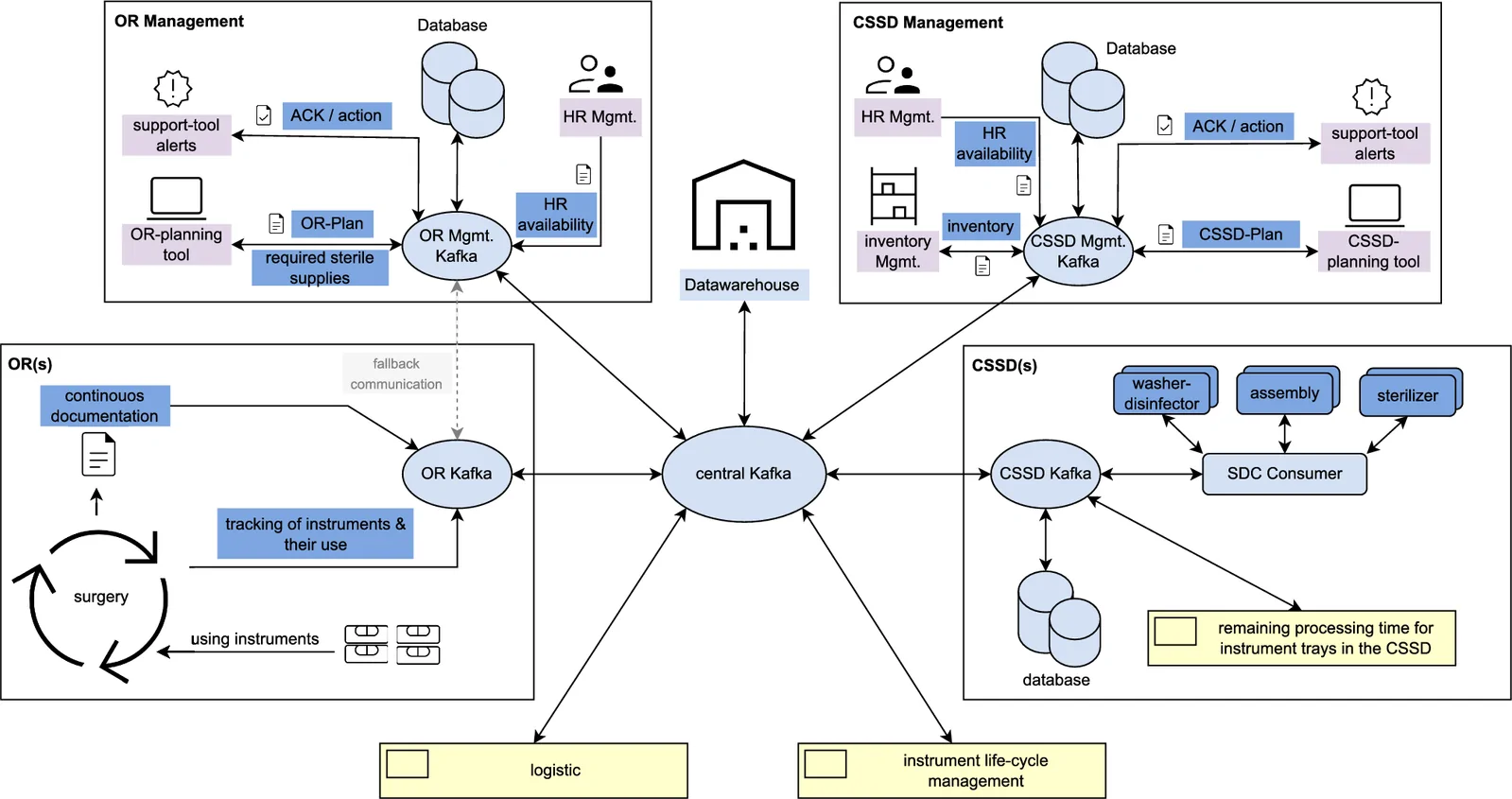
Overview of the system, showing layer assignments by colour: Application (purple), Knowledge (yellow), Gateway (light blue), Perception (dark blue); abbreviations: ACK=Acknowledgement, Mgmt.=Management

An overview of the proposed system is shown in Fig. 2.

## Discussion

This paper proposes a conceptual framework for integrating the CSSD into a broader smart hospital architecture. Its contribution lies in structuring requirements, actors, information flows, and candidate architectural principles for cross-domain integration. It does not provide a technical performance evaluation, a formal economic assessment, or a deployment-ready reference implementation. The following discussion therefore focuses on key trade-offs, limitations, and open questions that would need to be addressed in future implementation work.

### Single point of failure/systemic dependency

While Kafka clusters are designed for high availability, the central Kafka instance represents a conceptual single point of failure. In the event of its unavailability, inter-subsystem communication is disrupted, temporarily limiting access to globally shared support services, while local subsystem operations remain unaffected.

Certain subsystems, such as the OR management, rely on continuous inter-subsystem communication for efficient resource allocation. A fallback mechanism enabling direct system-to-system messaging preserves core functionality in cases of communication disruptions, albeit potentially without advanced support features.

Failures at the subsystem level can impair both internal and external messaging, limiting information propagation and updates to intelligent support services.

Failures at the subsystem level may likewise impair both internal and external information exchange, thereby restricting the propagation of updates to connected planning or decision support functions. Possible mitigation strategies include geographically distributed deployment, failover mechanisms such as automatic leader election and partition reassignment, and continuous monitoring of broker availability, message lag, and throughput [22]. In the present work, however, these measures are noted as potential design options rather than evaluated safeguards.

#### Latency and additional communication layer

The introduction of a communication layer, comprising subsystem-level and central Kafka instances, increases latency and governance overhead. Each message hop, serialisation process, and broker interaction contribute to increased end-to-end transmission times.

Latency requirements within smart hospital architecture are context-dependent. While strict real-time constraints exist in specific clinical scenarios, the proposed system primarily supports operational coordination, planning, and decision support in sterile goods processing and OR management. For these use cases, moderate latencies may be acceptable and could be offset by the benefits of decoupled, asynchronous communication.

Within the framework, Kafka is considered as a reference technology for event-driven integration because it supports decoupled communication, persistence, and scalable message exchange [22]. At the same time, cascading communication across multiple local and central instances may further increase propagation delays. The quantitative impact of such architectural decisions on latency, workflow performance, and decision support quality has not been evaluated in the present conceptual work and remains an important topic for future research.

#### Governance efforts

Establishing a shared, system-wide data backbone requires comprehensive governance to ensure a consistent data foundation across subsystems. This includes managing event schemes and data models for semantic consistency and evolution and implementing access controls. Although these governance efforts introduce additional organisational and operational complexity, they are essential for interoperability, traceability, and the scalable integration of heterogeneous subsystems.

Consequently, governance overhead should not be regarded solely as a drawback of the proposed architecture but as an inherent trade-off required to enable system-wide integration and coordinated decision support.

#### Consistency & data ownership

Due to the asynchronous, event-driven design, state changes within individual subsystems propagate through Kafka-based communication and may therefore become visible to other subsystems or central services with delays. This may result in temporary inconsistencies between local and global system views. However, for operational coordination, planning, and decision support, such delays might be acceptable and need to be further researched.

To handle the resulting eventual consistency in a controlled manner, each subsystem is designed to maintain a locally consistent state and acts as the authoritative source for its respective domain. Global system views are derived through asynchronous aggregation of subsystem-level events and are primarily used for coordination and decision support. The single source of truth is thus applied at the subsystem level, preserving autonomous operation.

### Security and privacy considerations

Security and privacy are critical for smart hospital architectures, particularly in distributed systems connecting multiple subsystems and organisational units. The proposed architecture processes and exchanges sensitive operational and personal data, such as information on resource allocation and process traceability, across subsystem boundaries. Maintaining data confidentiality and integrity is essential to prevent unauthorised access and external attacks.

Addressing these concerns requires security mechanisms, including authentication, authorization, access control, and secure communication channels, alongside privacy-preserving measures to handle sensitive information in compliance with regulatory requirements [13].

The presented architecture emphasises functional integration, interoperability, and system-level coordination. A detailed assessment of security and privacy, for example through threat modelling and evaluation of protective mechanisms, remains an important direction for future research.

### Communication standards

In the architecture, we propose the adaption of SDC to the SSC due to its demonstrated advantages in ORs and other hospital departments, achieving interoperability and semantic harmonisation [7]. Although SDC is used as the communication standard in the proposed architecture, the overall design is not tied to a specific technology. While alternative technologies, such as OPC UA, are feasible, the architecture is intentionally designed flexible, allowing SDC to be replaced or complemented by other communication standards. Consequently, the use of SDC does not limit the generalizability of the proposed approach across different communication technologies.

## Conclusion and future Work

This work presents a conceptual architecture for a resilient, smart SSC that addresses the structural fragmentation and reactive operation characterising current SSC practice. By treating the SSC as a safety-critical, data-driven production system and integrating it systematically with operating rooms and OR management, the proposed framework shifts SSC coordination from late, manual intervention towards proactive, information-driven control. Drawing on principles from smart manufacturing and leveraging open, service-oriented standards, the architecture establishes a coherent foundation for end-to-end visibility of instrument states, consistent data exchange, and coordinated decision support across departmental boundaries.

The proposed service-oriented, event-driven design enables modular integration of heterogeneous devices and systems while preserving local autonomy of subsystems. The consideration of IEEE 11073 SDC as a semantic connectivity layer, combined with a Kafka-based messaging backbone as a candidate integration mechanism, illustrates one possible approach to supporting data propagation, traceability, and future extensibility within such an architecture. In this way, the framework supports a transition from open-loop SSC operation to a more resilient, feedback-driven model that can better absorb variability in surgical demand without compromising safety or efficiency.

While the presented work remains conceptual, it identifies key architectural principles and design trade-offs related to latency, governance, and eventual consistency. Future work should focus on defining and prototyping functional modules that integrate into the proposed architecture. Building on the proposed messaging backbone, cross-cutting services (e.g. hospital-wide analytics, dashboards, decision support) could subscribe to SSC events via a central integration layer, while locally scoped modules (e.g. CSSD-specific tools) could interface with dedicated subsystem-level instances and consume a filtered subset of data. Future work should also assess the organisational and economic implications of the proposed framework, including implementation effort, governance overhead, and expected operational benefit under different hospital conditions. Further research will implement and test the concept in a dummy test system and, subsequently, in clinical test environments to identify practical challenges, risks, and feasibility constraints.
